# The Beat

**Published:** 2009-05

**Authors:** Erin E. Dooley

## Europe Puts EMFs on Alert

In April 2009 the European Parliament adopted a report urging the European Commission to set limits on how close mobile phone masts, antennas, high-voltage power lines, and other electromagnetic field (EMF)–transmitting devices can be to neighborhoods, schools, and health care facilities. The report asks the commission to review the scientific basis and accuracy of current EMF limits and recommends the annual publication of a map showing areas of EMF exposure, an annual report on EMF levels in Europe, labeling requirements to state EMF transmission levels of wireless devices, and increased education about the safest use and potential dangers of mobile phones, particularly for children and teenagers.

## First Bloom of Sister Study Findings

**Figure:**
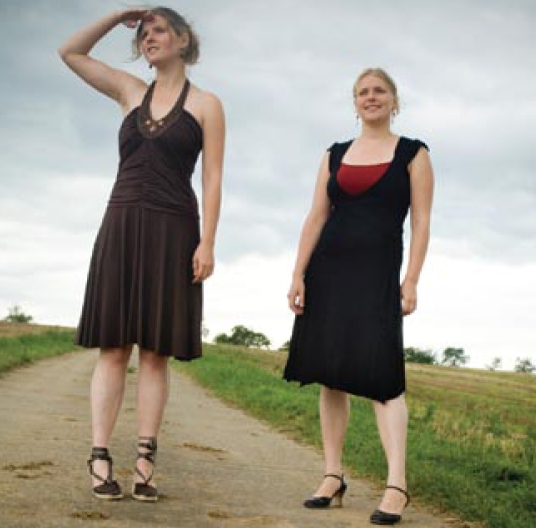
The Sister Study includes women whose sister had breast cancer.

Early findings from the NIEHS-sponsored Sister Study, published in the February and March 2009 issues of *Cancer Epidemiology Biomarkers & Prevention*, showed that women who maintained a healthy weight and perceived themselves to have less stress were less likely to have shorter telomeres, the repeating DNA sequences that protect the ends of chromosomes and help maintain genetic integrity during cell replication. Shortened telomeres are associated with a heightened disease risk and increased mortality rates for cancer and heart disease. These reports add to previous research showing that positive lifestyle changes can aid telomere activity.

## Water Certification in the Works

At the March 2009 World Water Forum, the Alliance for Water Stewardship announced a new labeling program that would state whether the water used to make a product came from a sustainable source. The initiative is similar to the Forest Stewardship Certifi cation, which labels wood products as sustainably harvested from certified lands. Alliance members hope to establish universal “core standards” for sustainable water management by mid 2009; from there, local agencies could add their own criteria to address regional needs. Within the next two decades, an estimated 3.9 billion people could live in drought-stricken areas, according to *OECD Environmental Outlook to 2030*, a 2008 report from the Organisation for Economic Co-operation and Development.

## Diacetyl Deadlock Broken?

Diacetyl, a chemical compound used to impart a buttery flavor to foods like microwave popcorn, may become hazardous when it is heated and inhaled over a long period. In exposed workers, it has been linked with bronchiolitis obliterans, a degenerative and potentially fatal lung disease. Regulation of worker exposure to diacetyl had been delayed by a Bush-era Advance Notice of Proposed Rulemaking, but in March 2009, Secretary of Labor Hilda Solis announced the withdrawal of the notice, which could allow OSHA to move forward more quickly with new regulations. The flavoring is deemed “generally regarded as safe” by the FDA; one case of bronchiolitis obliterans was identified in a consumer who ate two bags of microwave popcorn each day for several years.

**Figure:**
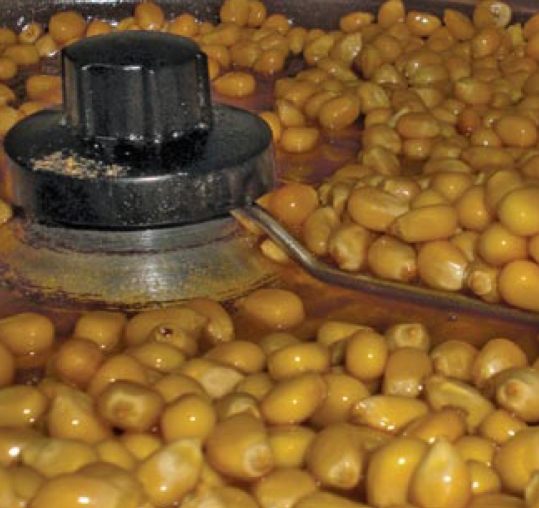
Some popcorn makers have stopped using diacetyl.

## New Gases on the Radar

At March’s Greenhouse 2009 meeting in Perth, Australia, researchers presented new information on two gases that have been linked with global warming for over a decade. Atmospheric levels of nitrogen trifluoride (which replaced perfluorocarbons in circuit board manufacturing) and sulfuryl fluoride (which replaced methyl bromide in pest control) are still low, but they are increasing rapidly—which is not unexpected, given their relatively recent introduction to the atmosphere. Controlling nitrogen trifluoride could be particularly key because it persists for hundreds of years in the atmosphere. The team called for the two gases to be added to future versions of the Kyoto Protocol.

## Climate Change Concerns Farmers

Across the globe, agricultural experts are calling on farmers to take climate change into account as they go about the business of feeding the world. In March 2009, the European Commission released a draft report warning that farmers in some regions of Europe may face disparities in crop production because of uneven effects of global warming across the region. To reduce emissions and prepare for climatic changes, the report endorses renewable energy and biotechnology, and also advocates a variety of organic soil management practices that help store carbon and are more resilient to climate fluctuations. In southern India, a women’s collective is already planting novel crop combinations, using fewer chemicals, and embracing other sustainable practices.

**Figure:**
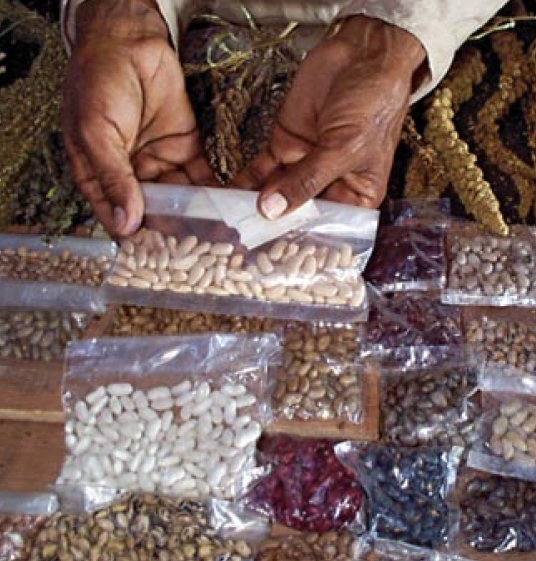
Growing a variety of crops in tandem can increase soil health.

